# Histone Demethylase KDM5B as a Therapeutic Target for Cancer Therapy

**DOI:** 10.3390/cancers12082121

**Published:** 2020-07-31

**Authors:** Anmi Jose, Gautham G. Shenoy, Gabriel Sunil Rodrigues, Naveena A. N. Kumar, Murali Munisamy, Levin Thomas, Jill Kolesar, Ganesha Rai, Praveen P. N. Rao, Mahadev Rao

**Affiliations:** 1 Department of Pharmacy Practice, Manipal College of Pharmaceutical Sciences, Manipal Academy of Higher Education, Manipal, Karnataka 576104, India; anmi.jose@learner.manipal.edu (A.J.); murali.m@manipal.edu (M.M.); levin.thomas@learner.manipal.edu (L.T.); 2 Department of Pharmaceutical Chemistry, Manipal College of Pharmaceutical Sciences, Manipal Academy of Higher Education, Manipal, Karnataka 576104, India; gautham.gs@manipal.edu; 3 Department of Surgery, Kasturba Medical College, Manipal Academy of Higher Education, Manipal, Karnataka 576104, India; gaby.rodrigues@manipal.edu; 4 Department of Surgical Oncology, Kasturba Medical College, Manipal Academy of Higher Education, Manipal, Karnataka 576104, India; naveenkumar.an@manipal.edu; 5 Department of Pharmacy Practice & Science, 567 TODD Building, 789 South Limestone Street, Lexington, KY 40539-0596, USA; jill.kolesar@uky.edu; 6 National Center for Advancing Translational Sciences, National Institutes of Health, 9800 Medical Center Drive, MSC 3370, Bethesda, MD 20892, USA; bantukallug@mail.nih.gov; 7 School of Pharmacy, Health Sciences Campus, 200 University Avenue West, University of Waterloo, Waterloo, ON N2L 3G1, Canada; praopera@uwaterloo.ca

**Keywords:** cancer, histone modification, inhibitor, KDM5B, molecular docking, repurposing

## Abstract

Lysine-specific demethylase 5B (KDM5B/PLU1/JARID1B) is found to be overexpressed in numerous malignancies, including breast, lung, skin, liver, and prostate cancer. Identification of molecules targeting the KDM5B enzyme could be a potential lead in cancer research. Although many KDM5B inhibitors with promising outcomes have been developed so far, its further application in clinical practice is limited due to toxicity and lack of target specificity. Here, we summarize the significance of targeting KDM5B in anticancer therapy and report the molecular docking studies of some known anti-viral agents, decitabine, entecavir, abacavir, penciclovir, and 3-deazaneplanocin A in the catalytic domain JmjC of KDM5B. These studies show the repurposing potential of identified anti-viral agents in cancer therapy.

## 1. Introduction

Studies over the past few decades and ongoing research reveal that, along with genetic alterations, epigenetic aberrations play a vital part in the evolution and progress of numerous diseases, including several types of cancers [1]. Epigenetic changes, including DNA methylation and histone modifications, cause altered gene expression without changing the DNA sequence [2,3].

Histone, being the functional protein, not only actively takes part in gene expression, but also acts as a spool for DNA to wind around. Any modification on histone molecules via methylation, acetylation, phosphorylation, ubiquitylation, or sumoylation alters the compact chromatin architecture, leading to abnormal gene expression [4]. Histone methylation on arginine or lysine, which are abundantly present on N- and C- terminal tails of histones, can either turn on or off transcription based on the degree and location of methylation [5].

Lysine-specific demethylation is finely tuned by two enzyme categories, called lysine-specific demethylase (KDM1/LSD1) and the JumonjiC (JmjC) domain, encompassing histone demethylases (KDMs). JmjC, being the largest among two families, displays high substrate specificity, with demethylase activity targeting histones H3 and H4 [6]. In mammals, around thirty KDMs have been identified and grouped as phylogenetically distinct subfamilies from KDM2-8 with numerous subcategories [7].

**Figure 1:**
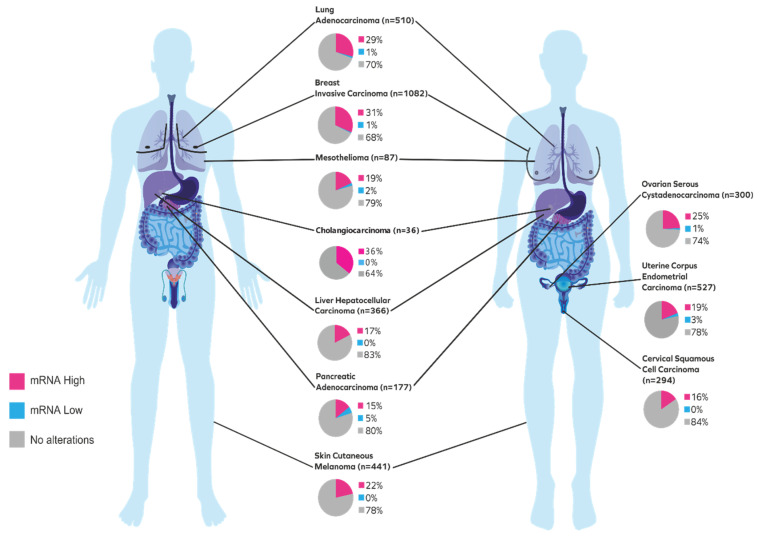
Pictorial representation of the top ten cancers with high KDM5B mRNA expression. Data extracted from the cBioPortal for cancer genomics—TCGA, PanCancer Atlas (*n* = number of patients).

The KDM5/JARID1 subfamily consists of four interrelated histone demethylase enzymes—KDM5A, KDM5B, KDM5C and KDM5D—involved in several human biological processes, including hormonal response, stem cell regeneration, genomic stability, cellular proliferation, and differentiation [8,9]. KDM5B (also known as PLU1 or JARID1B), demethylates lysine 4 of histone 3 (H3K4), and acts as a transcriptional repressor on certain tumor suppressor genes, thus converting it into the transcriptionally inactive state [10]. The deregulated expression of KDM5B has been implicated in numerous cancer types, including breast, lung, skin, prostate, testis, and liver [11]. Figure 1 depicts the top ten cancers, with high KDM5B mRNA expression data extracted from cBioPortal for cancer genomics—TCGA, PanCancer Atlas study [12,13,14]. Thus, KDM5B could be considered as a potential therapeutic target in precision oncology. Hence, drugs that can selectively inhibit KDM5B could be of substantial importance as a therapeutic intervention for such cancer types. This review attempts to comprehend, as thoroughly as possible, the oncogenic role of KDM5B in major cancer types and the drug repurposing potential of some known small molecules by targeting KDM5B’s catalytic domain through molecular docking studies.

## 2. Structure and Enzymatic Function of KDM5B

The JmjC family is reliant on Fe (II) and α- ketoglutarate co-factors for its demethylating activity. KDM5B gene consists of 4632 bases, positioned on chromosome 1, in the cytogenetic band of 1q32.1 with a molecular mass of 175 kilodaltons. It encodes a protein of 1544 amino acids [15] comprising an *N*-terminal Jumonji (JmjN) domain, a catalytic JmjC domain, an AT-rich domain (ARID), a C5HC2 zinc finger domain, a PLU1 motif and three plant homeobox domain (PHD1, PHD2, and PHD3). The domain architecture of KDM5B is highly conserved and homologous from yeast to humans. JmjN and JmjC are vital domains required for enzymatic activity, whereas AT-rich domain binds to GC rich DNA sequences. PHD domains contribute to substrate recognition in which it prevents reverse reaction [16,17,18,19,20,21]. ARID and PHD domains have very less influence, whereas JmjN and JmjC along with C5HC2 zinc finger domain have more impact on the KDM5B’s catalytic activity. The overall catalytic core is composed of three conserved domains consisting of: (i) the JmjN, JmjC, and ARID domains (residues 31–72, 375–602 and 94–100, respectively); (ii) a C-terminal helical domain (residues 604–671 and 737–753); (iii) a β-sheet composed of three β-strands (residues 673–734) that harbored a C5HC2 zinc finger motif [20].

## 3. Significance of KDM5B in Various Cancers

### 3.1. Breast Cancer

**Figure 2:**
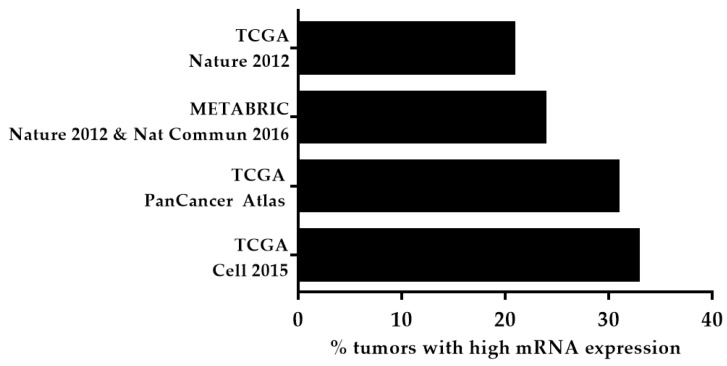
A graphical representation of the high KDM5B mRNA expression among breast cancer patients. Data extracted from the cBioPortal for cancer genomics.

Although the consistent expression of KDM5B in breast cancer was established by Lu P.J. et al. [22] in 1999, Yamane K. et al. in 2007 revealed the salient role of KDM5B in breast cancer cell proliferation via the transcriptional repression of tumor suppressor genes, including BRCA1 [19]. Subsequent studies both in vitro and in vivo, involving gene expression and KDM5B knockdown, confirmed its putative role in breast tumorigenesis [19,23,24,25]. One of the proposed mechanisms of KDM5B-mediated tumor cell proliferation was by repressing tumor suppressor miRNA let-7e [26]. Besides, the TFAP2C-Myc-KDM5B complex can repress p21, leading to tumorigenesis and therapy failure [27]. A recent study showed that estrogen receptor-positive (ER^+^) tumors, with KDM5B overexpression, had poor clinical outcomes and resistance to hormonal therapy [25]. Moreover, KDM5B expression was found to be negatively correlated with p16 protein expression [28]. Significantly, microRNA hsa-miR-448 can suppress KDM5B expression through MALAT1 and can prevent triple-negative breast cancer (TNBC) progression [29]. The downregulation of KDM5B led to 3′UTR lengthening of the cyclin D1 (CCND1) oncogene and lengthening of a tumor suppressor gene, DICER1, suggesting KDM5B as a novel target for 3′UTR processing [30]. KDM5B inhibition promoted the re-expression of tumor suppressor protein HEXIM1, and upregulated HEXIM1 aided in the inhibition of breast cancer cell proliferation using KDM5B inhibitors [31]. Recently, Paroni G. et al. showed that HER2–positive breast cancer cells were sensitive to KDM5 inhibition and KDM5 inhibitors exhibited a synergistic effect with HER2 targeting drugs, trastuzumab and lapatinib [32]. Similarly, numerous studies have reported the oncogenic role of KDM5B, where gene expression levels were related with poor prognosis, cancer cell proliferation, and metastasis [33,34,35]. Figure 2 shows the KDM5B mRNA overexpression in breast cancer from various studies retrieved from the cBioPortal for cancer genomics [12,13,14,36,37,38].

### 3.2. Lung Cancer

KDM5B expression rate was found to be highly elevated in neoplastic tissues, in contrast to normal tissues, irrespective of lung carcinoma histology [39,40,41]. The suppression of KDM5B expression showed a significant reduction in cancer cell growth via the E2F/RB1 pathway [40]. Han L. et al. reported in 2013 that KDM5B positively regulates brain metastasis in NSCLC [42]. Moreover, KDM5B aids in the proliferation, invasion, and metastasis activities of lung cancer cells through downregulated p53 [39]. Besides, Shen X. et al. reported that KDM5B overexpression positively correlated with size and stage of the tumor, lymph node metastasis, and reduced survival rate [39]. A recent study by Lu Y. et al. established that KDM5B plays crucial roles in hypoxia-induced gefitinib (EGFR TKI) resistance and epithelial–mesenchymal transition (EMT) [43].

### 3.3. Melanoma

The role of KDM5B in melanoma is still controversial. Roesch A. et al., in their studies, showed that KDM5B promoted tumor cell maintenance and metastasis, and its knockdown led to the exhaustion of melanoma cells [44]. However, KDM5B has also been suggested as a tumor-suppressive agent in melanocytic cells through the regulation of activities of the retinoblastoma protein [45,46]. Interestingly, it is evident that, in melanoma cells, KDM5B expression is dynamically regulated. However, KDM5B was overexpressed in certain benign tumors, whereas downregulated in aggressive and metastatic melanomas [47]. The relevance of KDM5B in chemo-resistance was confirmed when KDM5B depletion led to the increased sensitivity of melanocytes to anti-melanoma treatment [48].

### 3.4. Hepatocellular Carcinoma

KDM5B was often upregulated in hepatocellular carcinoma (HCC) samples, compared to corresponding non-neoplastic tissues. Besides, the gene expression level was associated with tumor properties and stages of cancer. HCC patients with KDM5B overexpression had more tumor metastasis, poor prognosis, and significantly shorter overall survival [49,50,51,52]. KDM5B exerts its oncogenic function by inactivating PTEN transcription [49] and by silencing KDM5B, which, remarkably, hinders cancer cell proliferation through p15 and p27 up-regulation [50].

### 3.5. Gastric Cancer

The aberrant expression of KDM5B mRNA was detected in gastric tumor specimens and cell lines in comparison with normal tissues. The expression rate also correlated with TNM stage and depth of invasion [53]. Various studies confirm the contribution of KDM5B in various signaling pathways for tumor cell development and migration [53,54]. Bao J. et al. reported that miR-194, which obstructs gastric cancer cell growth and invasion, directly targets and negatively regulates KDM5B [55]. These findings revealed the potential role of KDM5B, as a tumor promoter in gastric cancer. A recent study by Xu W. et al. described that increased KDM5B expression, associated with cisplatin resistance in gastric cancer cell lines and targeting KDM5B, reversed the phenotype [56].

### 3.6. Colorectal Cancer

The functional significance of KDM5B in colorectal cancer (CRC) stem cells was confirmed through in vitro and in vivo studies. KDM5B knockdown was found to surge H3K4 trimethylation at the p16/INK4A tumor suppressor’s promoter region, which subsequently induced cellular senescence [57]. Besides, KDM5B was found to be overexpressed in CRC tumor tissue compared with normal colon samples and this overexpression positively correlated with cancer progression [58].

### 3.7. Bladder Cancer

Knockdown and apoptosis studies demonstrated the close link of KDM5B, in cell cycle regulation and cancer cell maintenance, in bladder cancer cell lines. Overexpression of KDM5B was noted in the early and advanced stages of bladder cancer, irrespective of cancer grade [40]. A potential inverse connection between KDM5B and connexin 26 (CX26) in the progression of bladder cancer was reported, where KDM5B was found to be upregulated in contrast to CX26 [59].

### 3.8. Prostate Cancer

KDM5B was found to be overexpressed in prostate cancer tissues, compared to benign tissues, and has been suggested as a potential therapeutic target [15,60]. Various studies evaluated the role of microRNAs in regulating KDM5B. The forced miR-29a expression inhibited proliferation, and induced apoptosis in prostate cancer cells, by repressing KDM5B expression, whereas, by KDM5B targeting, miR-137 aided as a tumor suppressor in prostate carcinogenesis [61,62].

### 3.9. KDM5B in Other Cancers

KDM5B showed significantly high expression levels in patients with B-cell precursor acute lymphoblastic leukemia (B-ALL), compared to normal bone marrow [63]. The suppression of KDM5B hindered metastasis and the invasion of human oral squamous cell carcinoma and sensitized radiation therapy [64]. KDM5B overexpression was significantly associated with tumor cell proliferation in head and neck cancer, and KDM5B silencing caused cell growth suppression both in vitro and in vivo [65]. Similar studies supporting the oncogenic role of KDM5B were observed in other malignancies, such as esophageal squamous cell carcinoma [66], cervical cancer [67], renal cell carcinoma [68], epithelial ovarian cancer [69] and neuroblastoma [70].

### 3.10. KDM5B as a Therapeutic Target

Apart from the oncogenic and tumorigenic role, KDM5B is also considered as a cancer-testis antigen (CTA), where gene expression is restricted in normal tissues except adult testes [22,71]. Therefore, abnormal overexpression of KDM5B in any neoplastic tissue, in comparison with corresponding non-neoplastic tissues, makes it an ideal therapeutic target. Furthermore, CTA properties suggest that targeting KDM5B may be associated with reduced toxicity. Therefore, the development of specific KDM5B inhibitors will offer a deeper insight into the therapeutic potential for many types of cancers.

**Figure 3:**
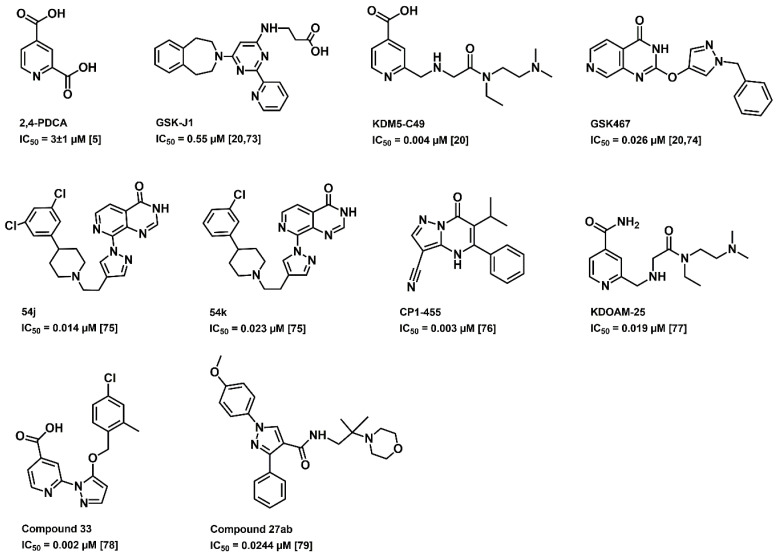
Previously reported KDM5B inhibitors (IC_50_: half maximal inhibitory concentration).

As Fe (II) and α- ketoglutarate are the two vital co-factors of KDM5B in demethylation, studies are focusing on the development of compounds that mimic α-ketoglutarate or compounds that chelate with Fe (II). Over the years, many KDM5B inhibitors have been developed (Figure 3) and have provided promising outcomes [72]. The 2,4-pyridinedicarboxylic acid (2,4-PDCA) inhibits KDM5B in vitro with an IC_50_ value of 3 ± 1 µM [5], whereas GSK-J1 with an IC_50_ of 0.55 µM [20,73]. GSK467 displayed an inhibitory concentration of 0.026 µM. The crystalline structure of KDM5B with GSK467 provides a possible template for the development of selective KDM5B inhibitors [20,74]. Compounds 54j and 54k are potent, cell permeable dual inhibitors of the KDM4 and KDM5 subfamilies. Compounds 54j and 54k inhibit KDM5B with IC_50_ values 0.014 µM and 0.023 µM, respectively, and have shown adequate cellular permeability [75]. CPI-455 with IC_50_ of 0.003 µM, was identified as a pan-KDM5 inhibitor that competitively binds to the two cofactor binding sites [76]. KDM5-C49, a 2,4-PDCA analogue, and further modified KDOAM-25 have shown acceptable inhibitory potency on KDM5B, in which KDOAM-25 exhibited good stability, high selectivity and low cytotoxicity [20,77]. An orally available, potent inhibitor of KDM5B, named compound 33, was identified through structure activity relationship exploration and presented promising selectivity compared to early inhibitors [78]. Recently, a pyrazole derivative, compound 27ab, was discovered, which inhibited MKN45 cell proliferation, wound healing and migration [79]. All these inhibitors are in their initial stage of development and have not made it to the clinics yet.

## 4. Molecular Docking Studies

**Figure 4:**
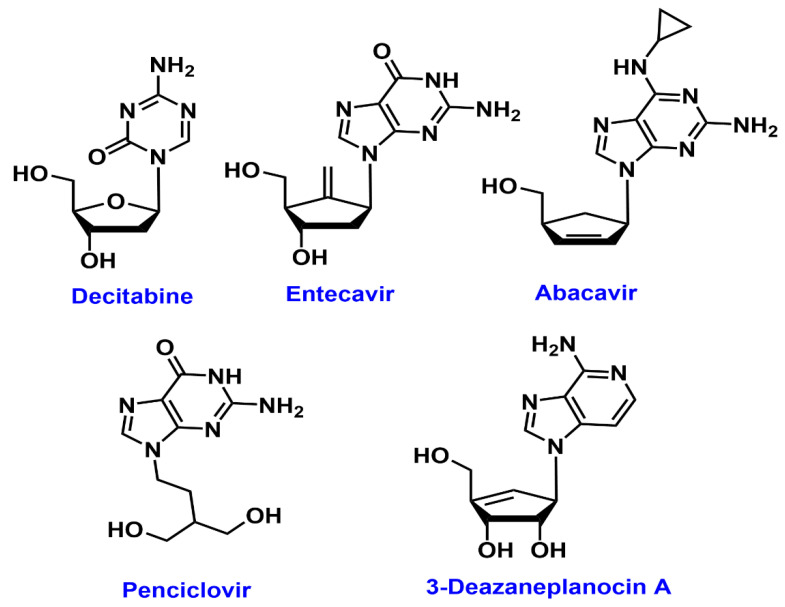
Selected antiviral drugs targeting KDM5B.

The JmjC domain of native KDM5B, which is involved in histone demethylation, contains Fe (II) ion and α-ketoglutarate as cofactors. This site is an attractive target to develop KDM5B inhibitors. One of the solved crystal structures of KDM5B, with an inhibitor GSK467 in the JmjC domain, contains Mn (II) instead of Fe (II) [20]. This structure provides an excellent starting point to develop novel KDM5B inhibitors using computational tools. Accordingly, we used computational modeling to explore the potential of known drugs to inhibit KDM5B. Drug repurposing is an approach where new therapeutic indications for already marketed drugs are discovered. This approach aims to lessen the price and time involved in drug discovery process [80,81]. On this note, we considered the drug repurposing potential of nucleoside derivatives, which are well known as antiviral agents [82]. Several studies in the past have shown that nucleoside derivatives, with monocyclic or bicyclic ring systems, exhibit anticancer properties [83,84,85]. We investigated the drug repurposing potential of known compounds, such as decitabine, entecavir, abacavir, penciclovir, and 3-deazaneplanocin A (DZNep) (Figure 4), which are all nucleoside derivatives, as inhibitors of KDM5B, by conducting molecular docking experiments. In addition, we compared the binding modes of previously reported KDM5B inhibitors containing planar bicyclic rings, such as GSK467, 54j, and CPI-455, with penciclovir and DZNep, to determine their interactions in the JmjC domain of KDM5B. It should be noted that decitabine (Dacogen^®^) is currently used to treat myelodysplastic syndrome (MDS), entecavir (Baraclude^®^) is used as an anti-hepatitis B agent, abacavir (Ziagen^®^) is used to treat HIV infections and penciclovir (Denavir^®^) is a known drug used in the treatment of herpes infections. The carbocyclic adenosine derivative DZNep, is a known inhibitor of histone methyltransferase EZH2 and is a promising compound in cancer immunotherapy [86]. Molecular docking studies were carried out using the computational software, Discovery Studio (DS) Structure-Based-Design (BIOVIA Inc, Dassault Systemes, USA). Moreover, previous studies have shown that nucleoside derivatives with either a monocyclic or bicyclic ring exhibit anticancer property. The ligands decitabine, entecavir, abacavir, penciclovir, and DZNep were built in 3D, using the Small Molecules module in DS using CHARMm force field. The X-ray crystal structure of human KDM5B was obtained from PDB (pdb id: 5FUN) [20]. This KDM5B structure contains a pyridopyrimidine ligand GSK467 and the catalytic site contains Mn^2+^ ion. All the water molecules were removed and a 10 Å binding site sphere was defined by selecting the bound ligand GSK467, after which it was deleted. The KDM5B protein was prepared using the Macromolecules module in DS using CHARMm force field. Docking was carried out using the LibDock algorithm by employing 100 hotspots, a docking tolerance of 0.25 Å, and an implicit solvent model, with a distance-dependent dielectric constant and CHARMm force field. The binding poses obtained, were further energy minimized using Smart minimizer (1000 steps and an RMS gradient of 0.001 kcal/mol) and ranked using the LibDock scoring function. The ligand-binding interactions were further studied by considering the polar and nonpolar contacts in the KDM5B binding site. The general computational methods applied were as per our previously reported method [87].

**Figure 5:**
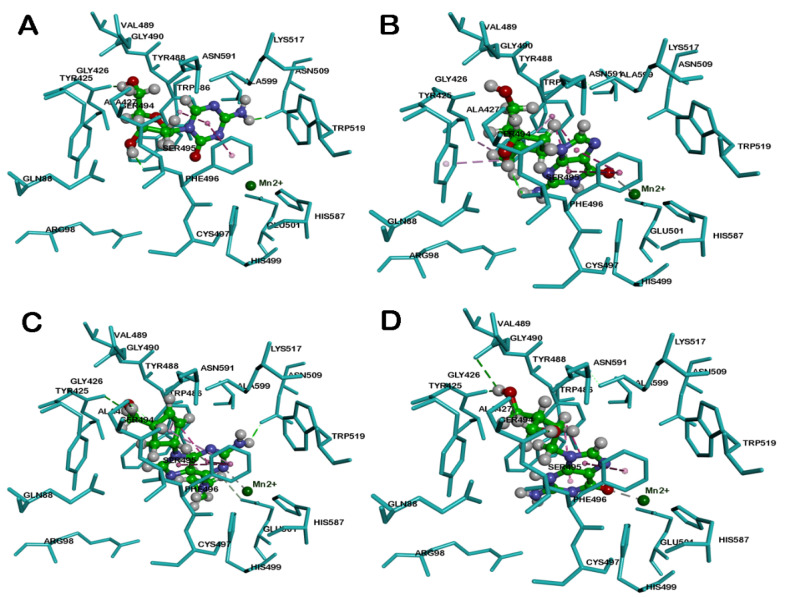
The binding modes of decitabine (**A**), entecavir (**B**), abacavir (**C**) and penciclovir (**D**) in ball and stick cartoon within the JmjC domain of human KDM5B (pdb id: 5FUN). Mn^2+^ ion is shown as a green circle. Hydrogen atoms are removed to enhance clarity. Polar and nonpolar interactions are color-coded.

Docking the nucleoside decitabine in the KDM5B catalytic site shows that the 6-member azacytidine ring was in the α-ketoglutarate binding pocket and underwent π–π stacked hydrophobic interactions with aromatic rings of Tyr488 and Phe496, respectively (distance < 4.1 Å). The azacytidine N3 was not forming any metal-ligand bonding with Mn^2+^ center (distance ≈ 5.0 Å, Figure 5, Panel A). The deoxy sugar moiety was in a region comprised of Tyr425, Gly426, and Ser495. The hydroxy and the hydroxymethyl groups were in contact with Ser495 and Gly426 backbones, via hydrogen bonding interaction (distance < 1.9 Å, Figure 5, Panel A). These studies suggest that decitabine can undergo favorable interactions with KDM5B.

Docking entecavir in KDM5B shows that the bicyclic guanosine ring was oriented in the α-ketoglutarate binding pocket, where it underwent some favorable interactions. The pyrimidine and imidazole rings were in contact with Tyr488 and Phe496 via four π–π stacked hydrophobic interactions (distance < 4.5 Å, Figure 5, Panel B). Interestingly, the guanosine C6 ketone (C = O) underwent metal–ligand bonding with the Mn^2+^ center (distance = 2.6 Å). The exocyclic alkene was in contact with Tyr425, Ala427, and Tyr488 (distance < 5.0 Å), whereas the hydroxy group of the cyclopentenyl ring formed a hydrogen bond with the backbone of Ser495 (distance < 2.0 Å, Figure 5, Panel B). In general, entecavir exhibited better binding interactions with KDM5B compared to decitabine. This can be attributed to the presence of a planar bicyclic guanosine ring in entecavir, and the metal–ligand bond with the guanosine ketone.

Docking the anti-HIV agent abacavir with KDM5B shows that it was buried in the α-ketoglutarate binding pocket closer to the metal center similar to entecavir, and the purine ring underwent π–π stacking interactions with aromatic rings of Tyr488 and Phe496 (distance < 4.8 Å) and the N1 of purine ring was in contact with Mn^2+^ (distance = 2.6 Å). Furthermore, the C2 amino substituent underwent hydrogen bonding interaction with the backbone of Asn509 (distance < 1.9 Å, Figure 5, Panel C). The cyclopentenyl ring was in contact with Tyr488 and Phe496 via hydrophobic interactions (distance < 4.8 Å).

Docking another purine-based drug penciclovir, which contains a guanosine ring similar to entecavir in the KDM5B, shows that it was exhibiting a similar binding mode as entecavir, and the guanosine ring was in hydrophobic contact with Tyr488 and Phe496 by forming several π–π stacking interactions (distance < 4.4 Å, Figure 5, Panel D). The guanosine ketone forms a metal–ligand interaction with Mn^2+^ (distance = 2.4 Å). The acyclic sugar moiety was closer to a polar region comprised of Gly426, Ser494, Val489, and Asn591. The hydroxy and hydroxymethyl substituents form hydrogen-bonding interactions with Gly429, Ser494, and Val489 backbones (distance < 2.8 Å, Figure 5, Panel D). These studies show that drugs containing a bicyclic purine template exhibit favorable binding in KDM5B.

**Figure 6:**
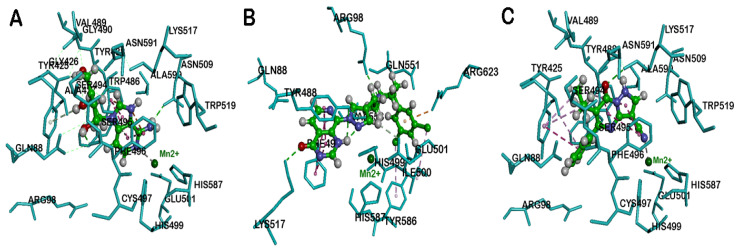
The binding mode of DZNep (**A**), 54j (**B**) and CPI-455 (**C**) (ball and stick cartoon) within JmjC domain of human KDM5B (pdb id: 5FUN). Mn^2+^ ion is shown as a green circle. Hydrogen atoms are removed to enhance clarity. Polar and nonpolar interactions are color-coded.

Docking DZNep, an investigational drug molecule, which also contains a planar bicyclic imidazo[4,5-c]pyridine ring template (Figure 4), and a cyclopentenyl moiety, shows that the bicyclic imidazo[4,5-c]pyridine ring, forms a π–π stacking interaction with Tyr488 and Phe496 (distance < 4.3 Å). Interestingly, the pyridine nitrogen, chelated with Mn^2+^ (distance = 2.8 Å, Figure 6A) and C2 amino substituents formed a hydrogen bond interaction with backbone of Asn509. The cyclopentenyl hydroxyl and hydroxymethyl substituents also underwent hydrogen bonding interactions with Gly426 and Ser495 (distance < 2.0 Å).

Among these compounds, DZNep exhibited the most favorable binding interaction with KDM5B. The binding interaction was measured by calculating the LibDock scores for the best binding poses. The ranking order was DZNep (LibDock score = 131.60) > penciclovir (LibDock score = 129.84) > entecavir (LibDock score = 121.26) > abacavir (LibDock score = 118.10) > decitabine (LibDock score = 109.55). Molecular docking studies of known KDM5B inhibitor, 54j (Figure 6B), shows that its bicyclic pyridopyrimidine ring underwent van der Waal’s contact with Tyr488 and Phe496 (π–π stacked interactions, distance < 5 Å) and the pyrimidinone ketone formed a hydrogen bond with the side chain of Lys517 (distance = 1.55 Å). Interestingly, the nitrogen of pyrazole and pyrimidinone rings was close to the metal center, indicating the ability of 54j to form metal–ligand interactions (Figure 6B). The piperidine benzene with 3,5-dichloro substituents, was oriented in a hydrophobic pocket made up of Ile500, Val553 and Tyr586. Furthermore, 54j is a much larger molecule (molecular volume = 345.7 Å^3^) compared to GSK467, CPI-455, decitabine, entecavir, abacavir, penciclovir, and DZNep (molecular volume range ~ 168.7–231.5 Å^3^). Therefore, 54j is able to bind in the entire span of the JmjC domain (Figure 6B), which is not possible for the antivirals investigated in this study, as they are smaller. This is also supported by a superior LibDock score obtained for 54j (LibDock score = 145.08). However, similar to 54j, all the bicyclic ring-containing agents, entecavir, abacavir, penciclovir, and DZNep, are able to undergo metal–ligand interaction (Figure 5 and Figure 6), which is a common feature observed.

Molecular docking studies of the known KDM5B inhibitor, CPI-455 (Figure 6C), shows that it was oriented in a similar region compared to molecules containing the planar bicyclic ring investigated in this study. The bicyclic pyrazolopyrimidinone ring of CPI-455 was in van der Waal’s contact with Tyr488 and Phe496 (distance < 5 Å), and the pyrimidinone ketone underwent a hydrogen-bonding interaction with the Lys517 side chain (distance = 2.48 Å). The C6 isopropyl substituent was in a hydrophobic pocket and underwent multiple interactions with Tyr425 and Ala427 (distance < 5 Å), whereas the C5 phenyl ring underwent π–π stacking interactions with Tyr425 (distance < 5 Å). Interestingly, the C3 nitrile substituent was able to chelate with the metal center (distance = 2.6 Å, Figure 6C) which shows the significance of a nitrile substituent in KDM5B binding (LibDock score = 120.19). These molecular docking studies also show that bicyclic ring-containing agents (e.g., 54j, CPI-455, entecavir, abacavir, penciclovir, and DZNep), exhibit better binding in the JmjC domain of KDM5B, compared to monocyclic ring-containing decitabine, which is also reflected in a lower LibDock score (109.55) obtained for decitabine compared to other molecules.

**Figure 7:**
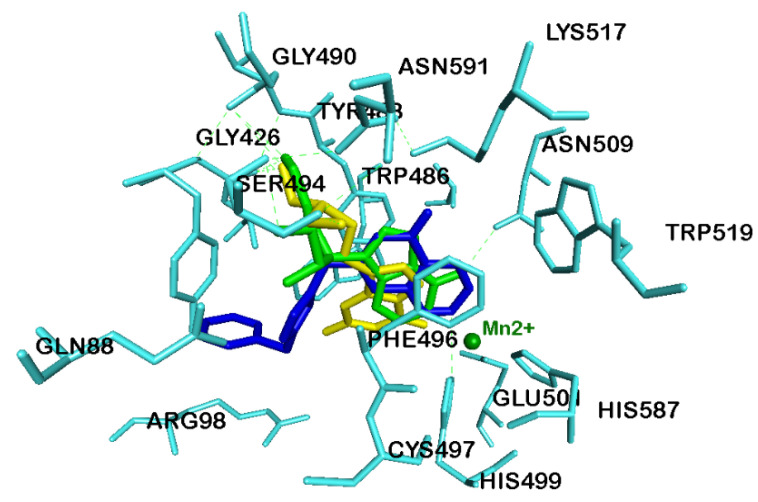
Comparison of binding modes of GSK467 (blue stick cartoon), penciclovir (yellow stick cartoon) and DZNep (green stick cartoon) in the JmjC domain of human KDM5B (pdb id: 5FUN). Mn^2+^ ion is shown as a green circle. Hydrogen atoms are removed to enhance clarity.

The binding modes of DZNep and penciclovir, which exhibited superior LibDock scores, were compared with the known X-ray structure of GSK467 (Figure 3), bound to KDM5B. This investigation shows that, similar to GSK467, the planar bicyclic rings of DZNep and penciclovir were oriented in the α-ketoglutarate binding pocket, and more significantly, the pyridine nitrogen of DZNep and guanosine ketone of penciclovir were in contact with the metal center Mn^2+^ (Figure 7). These observations were similar to the binding mode of GSK467 in the JmjC domain of human KDM5B, where the pyridopyrimidine nitrogen interacts with the metal center (Figure 7) [20].

These results also show that small molecules, which possess planar bicyclic heterocyclic rings, exhibit favorable binding toward the JmjC domain of human KDM5B. This is due to their ability to undergo interactions with the catalytic site metal ion, which is an important criterion in KDM5 inhibition and is consistent with previous studies [20,77,88]. Our molecular docking investigations show that decitabine, entecavir, abacavir, penciclovir, and DZNep have the potential to be repurposed as KDM5B inhibitors to treat various types of cancers.

## 5. Conclusions

The leading role of KDM5B oncogene in tumorigenesis and cancer progression is well established in various malignancies and is therefore, considered as a potential therapeutic target. Although many small molecule inhibitors of KDM5B have been identified so far, their possible clinical application in cancer treatment is a work in progress. This study provides a rationale on how repurposing antiviral drugs could contribute to the discovery and development of KDM5B inhibitors. We anticipate that these studies will stimulate further research in considering drug repurposing approaches to target KDM5B, and to discover novel therapies for various cancers.
